# Prognostic significance of semi-quantitative FDG-PET parameters in stage I non-small cell lung cancer treated with carbon-ion radiotherapy

**DOI:** 10.1007/s00259-019-04585-0

**Published:** 2019-11-22

**Authors:** Suman Shrestha, Tetsuya Higuchi, Katsuyuki Shirai, Azusa Tokue, Shreya Shrestha, Jun-ichi Saitoh, Hiromi Hirasawa, Tatsuya Ohno, Takashi Nakano, Yoshito Tsushima

**Affiliations:** 1 Department of Diagnostic Radiology and Nuclear Medicine, Gunma University Graduate School of Medicine, 3-39-22 Showa, Maebashi, Gunma 371-8511 Japan; 2 Department of Diagnostic Radiology and Nuclear Medicine, Nepal Cancer Hospital and Research Center, Harisiddhi, Lalitpur, Nepal; 3 Gunma University Heavy Ion Medical Center, Gunma University, Maebashi, Japan; 4 Department of Nephrology and Rheumatology, Gunma University Graduate School of Medicine, Maebashi, Japan; 5 Research Program for Diagnostic and Molecular Imaging, Division of Integrated Oncology Research, Gunma University Initiative for Advanced Research (GIAR), Maebashi, Japan

**Keywords:** Stage I non-small cell lung cancer (NSCLC), Carbon-ion radiotherapy (C-ion RT), ^18^FDG PET/CT, Metabolic tumor volume (MTV) histology, Prognostic factor

## Abstract

**Purpose:**

Prognostic significance of volumetric ^18^F-fluorodeoxyglucose (FDG) positron emission tomography/computer tomography (PET/CT) parameters in carbon-ion radiotherapy (C-ion RT) treated stage I non-small cell lung cancer, and need of histology-wise separate cut-off values for risk stratification were assessed.

**Methods:**

Thirty-nine patients (29 men and 10 women, 71.9 ± 8.3 years) who underwent FDG PET/CT examinations before C-ion RT were retrospectively evaluated. FDG-PET parameters: standardized uptake values (SUVmax, SUVpeak, and SUVmean), metabolic tumor volume (MTV), and total lesion glycolysis (TLG), and clinicopathological variables were assessed for prognosis using Cox proportional hazards regression analysis. Mann-Whitney test compared medians of significant parameters between adenocarcinoma (AC) and squamous cell carcinoma (SCC), and Kaplan-Meier curves were plotted for median-based low- and high-risk groups.

**Results:**

Median follow-up period was 44.8 months. 1/2/3-year overall survival (OS), progression-free survival (PFS) and local control (LC) rates were 94.9/84.3/70.8, 82.1/69.2/58.4 and 97.3/85.7/82.3%. Multivariate analysis revealed age (hazard ratio, HR: 1.09; 95% confidence interval, CI: 1.0–1.19, *p* < 0.05) and MTV (HR 4.83, 95% CI 1.21–19.27, *p* < 0.03) predicted OS, and only MTV predicted PFS (HR 5.3, CI 1.32–21.35, *p* < 0.02) independently. Compared with AC, SCC had higher MTV (median, 6.625cm^3^ vs 0.2 cm^3^, *p* < 0.01). Single MTV cut-off based on overall cohort was insignificant in SCC for PFS (*p* > 0.02); separate cut-offs of MTV, 0.2 cm^3^ for AC (*p* < 0.03) and 6.625 cm^3^ for SCC (*p* < 0.05) were relevant.

**Conclusion:**

Among all FDG PET/CT parameters, only MTV beared prognostic ability for stage I NSCLC treated with C-ion RT, and its histological variation may need consideration for risk-adapted therapeutic management.

## Introduction

Stage I non-small cell lung cancer (NSCLC) accounts for 15–20% of total NSCLC cases and surgery is the mainstay of treatment. Due to unwillingness or inoperability because of difficult tumor sites, radiotherapy like stereotactic body radiation (SBRT) is considered alternative standard therapy. Recently, carbon-ion radiotherapy (C-ion RT) has emerged as an alternative therapeutic option and is currently practiced in a few centers of the world [1–4].

C-ion RT is expected to have better prognosis because of its high-focused beams to the cancer cells due to distal fall-off of Bragg peak, less lateral scatter, and double-stranded DNA breaks. However, still recurrences or metastases are not so rare. Prognostic studies and accordingly treatment planning are required to prevent disease progression [4–6].

TNM staging is a widely accepted prognostic tool. However, in stage I, there are no lymph nodes and distant metastasis, so it is unable to explain the wide variation in survival outcomes within same stage, depending on the tumor size alone. Recently, positron emission tomography/computer tomography (PET/CT) using 18-fluorine deoxyglucose (^18^F-FDG) has been reported to be a very useful tool in diagnosing, staging, and treatment planning of NSCLC [7]. Different FDG PET/CT parameters like maximum standardized uptake value (SUVmax) and volumetric parameters like metabolic tumor volume (MTV) and total lesion glycolysis (TLG) have been reported to be significant prognostic factors in stage I NSCLC treated with various therapies, however, the results are conflicting [8–10].

In case of C-ion RT, only SUVmax has been studied so far in heavy ion department of our university [11], but histological variation in the FDG PET/CT parameters was not taken into account; it has been reported that the glucose metabolism differs in different histologies [12]. Clinically diagnosed patients without histology reports were also included in previous study [11] and follow-up was only about 2 years. Multivariate analysis was not performed and methods seemed inappropriate to draw solid conclusions. Thus, a detailed analysis using accurate methods was deemed necessary. We hypothesized that volumetric FDG PET/CT parameters would be superior to SUVmax in survival prognosis in NSCLC stage I patients treated with C-ion RT and there would be histology-wise variation in FDG PET/CT parameters.

Here, we aimed to evaluate the prognostic significance of pretreatment volumetric FDG PET/CT parameters and to determine whether histological variation needs to be considered or not in stage I NSCLC patients undergoing C-ion RT.

## Materials and methods

### Patient eligibility

In this study, we retrospectively enrolled the patients who were treated with C-ion RT between June 2010 and October 2016 in our institution with the following inclusion criteria: (1) stage I NSCLC with measurable lung lesions on FDG PET/CT, (2) FDG PET/CT done in our university hospital before C-ion RT, (3) confirmed pathology of the tumors, (4) follow-up period > 6 months, and (5) no any other anti-cancer therapy before C-ion RT.

This study was approved by the institutional review board of our institution and the informed consent of the patients was waived because of its retrospective nature. Electronic medical records were reviewed and we found 133 cases of NSCLC who underwent C-ion RT in that duration. Out of the 60 patients undergoing FDG PET/CT, 21 patients did not meet the other inclusion criteria: two had previous therapy, two had stage IIA, one had stage IIB, two had IIIA cancers, two had just 1-month follow-up, and 12 were clinically diagnosed cases without pathology reports. Hence, the remaining 39 patients were enrolled in this study. The tumor staging was determined by enhanced CT, brain magnetic resonance imaging (MRI), and FDG PET/CT.

### C-ion RT

Application of C-ion RT technique and treatment planning were done as described previously [13]. Heavy particle accelerator at our institution generated carbon-ion beams of 290, 380, and 400 MeV. After stabilizing the patient on fixation cushions and thermoplastic shells, CT simulation was done and delineation of gross tumor volume (GTV) was performed on visible lesions on lung window CT images. To include the subclinical disease extension, in addition to GTV, 5–8 mm margin within the lung parenchyma was set for clinical target volume. The planning target volume consisted of the set-up margin, and the internal margins obtained by using tumor motion of four-dimensional CT images. Dose of the C-ion RT was calculated as per the tumor sub-stage; 52.8 Gy (RBE) for T1a-b and 60Gy for T2a, and each patient received C-ion RT in four fractions within a week.

### FDG PET/CT

All patients had obtained FDG PET/CT scans before initiating C-ion RT. Following 6 h of fasting and confirming the blood glucose level to be less than 150 mg/dL, intravenous FDG injection was administered with a maximum activity of 400 MBq and PET scans were acquired 60 min after injection, using a PET/CT scanner (Discovery STE, GE Healthcare, MW) with a 700-mm field of view. Four to 10 bed positions (3-min acquisition in each bed position) were acquired depending on the range of imaging. Transverse images of FDG after attenuation correction were reconstructed with the ordered-subsets expectation-maximization algorithm into 128 × 128 matrices with a slice thickness of 3.27 mm.

Two experienced nuclear physicians reviewed the FDG images blindly and used syngo.via software (Siemens Medical Solutions, Erlangen, Germany) for calculation of PET parameters. Two observers calculated the parameters twice at an interval of around 1 month between two readings independently and they were blinded to the clinical outcomes. A region of interest (ROI) was drawn around the primary lesion with boundaries drawn to include the lesion in transaxial, coronal, and sagittal views. Standard uptake values (maximum SUV (SUVmax), peak SUV (SUVpeak), and mean SUV (SUVmean)) were obtained. SUV was defined as the following: SUV*x* = activity concentration in the ROI (*x*)/(injected dose/bodyweight of the patient); in SUVmax, *x* = max, i.e., single hottest voxel referring to the maximum activity in the ROI; in SUVpeak, *x* = peak, i.e., mean value of the voxels within sphere of 1-cm radius around the hottest voxel; and in SUVmean, *x* = mean, i.e., mean value of the voxels within the ROI. Volumetric parameters like metabolic tumor volume (MTV) and total lesion glycolysis (TLG) were also assessed. MTV was calculated by using an SUV of 2.5, reflecting the sum of all voxels with SUV value of 2.5 or more. TLG was calculated as MTV multiplied by the average SUV of 2.5.

Every 3 months for the first year and then every 6 months, patients were followed-up clinically and by CT examination. FDG-PET/CT was performed 2 months after the treatment and then every year. For any increment in the tumor size noted in CT scan, FDG PET/CT was performed. If FDG accumulation was observed, it was regarded as local recurrence and was further confirmed pathologically by biopsy, cytology, or salvage surgeries. Common Terminology Criteria for Adverse Events version 5.0 was used to score the toxicities, including pneumonitis, dermatitis, esophagitis, and chest wall pain [14].

### Statistical analysis

Overall survival (OS), progression-free survival (PFS), and local control (LC) rates were calculated by Kaplan-Meier estimator method. OS was defined as the time from the first day of the C-ion RT till the day of death or last follow-up. PFS was calculated as the time from the first day of the C-ion RT to the day of recurrence or metastasis. LC referred to the time from the first day of the C-ion RT to the day of local recurrence.

Survival analysis was carried out by the Cox-proportional hazard regression model—unilateral and multivariate analysis to know the impact of PET parameters and clinical variables (age, sex, tumor size, histology, operability, and radiation dose). Kaplan-Meier curves with log-rank test were used to plot the low- and high-risk groups of significant factor for survival outcomes. The groups were dichotomized based on median. Mann-Whitney test was performed to evaluate the differences in the uptake values in sub-groups based on histology. The Kaplan-Meier curve with log-rank test was also used in adenocarcinoma (AC) and squamous cell carcinoma (SCC), for evaluating low- and high-risk groups of significant parameter, stratified by median. Accuracy of the predictive performance of the parameters was analyzed by area under the curve (AUC) in receiver operating characteristic (ROC) curve analysis.

Intraobserver and interobserver variabilities between the two observers were calculated using correlation and regression analyses evaluating different aspects of the agreement. All statistical analyses were two-sided with *p* value < 0.05 set as statistically significant and performed using SPSS software version 25 (IBM Corp., Armonk, NY).

## Results

### Patients’ characteristics

Thirty-nine patients (29 men and 10 women; age 71.8+/− 8.3 years old, range 54–85) were enrolled in this study (Table 1). The median interval between the diagnostic FDG PET/CT scan and the start of C-ion RT was 21 days (range, 9–96). The median follow-up period was 44.8 months (range, 8.9–83.8). Fourteen patients (36%) were dead at the time of analysis. Histopathological diagnoses were AC in 23 patients (59%) and SCC in 16 (41%).

**Table 1:** Demographic Data in 39 patients with Stage I NSCLC Treated with Carbon ion therapy

Parameter	Patients (*n* = 39)	Frequency (%)
Age(y)*	73 (53–85)	N/A
Follow-up period (mo)*	44.8 (8.9–83.8)	N/A
Sex		
Male	29	74.4
Female	10	25.6
ECOG performance status		
0	15	38.5
1	22	56.4
2	02	0.1
Operability		
Operable	27	69.2
Inoperable	11	28.2
Unknown	01	2.6
Histologic nature of tumor		
Adenocarcinoma	23	58.9
Squamous cell carcinoma	16	41.1
TNM stage		
T1aN0	14	35.9
T1bN0	15	38.5
T2aN0	10	25.6
Overall stage		
Stage IA	29	74.4
Stage IB	10	25.6
Tumor size (mm)*	22 (8.2–48)	N/A
Location**		
Central	13	33.3
Peripheral	26	66.7
Irradiation dose (Gy)		
52.8	26	66.7
60	13	33.3

N/A – not applicable; * Data are medians, with ranges in parentheses.

** Lesions within 2 cm of the bronchial tree or mediastinal structures were considered to be central; all others were considered to be peripheral

### OS, PFS, and LC

**Fig. 1:**
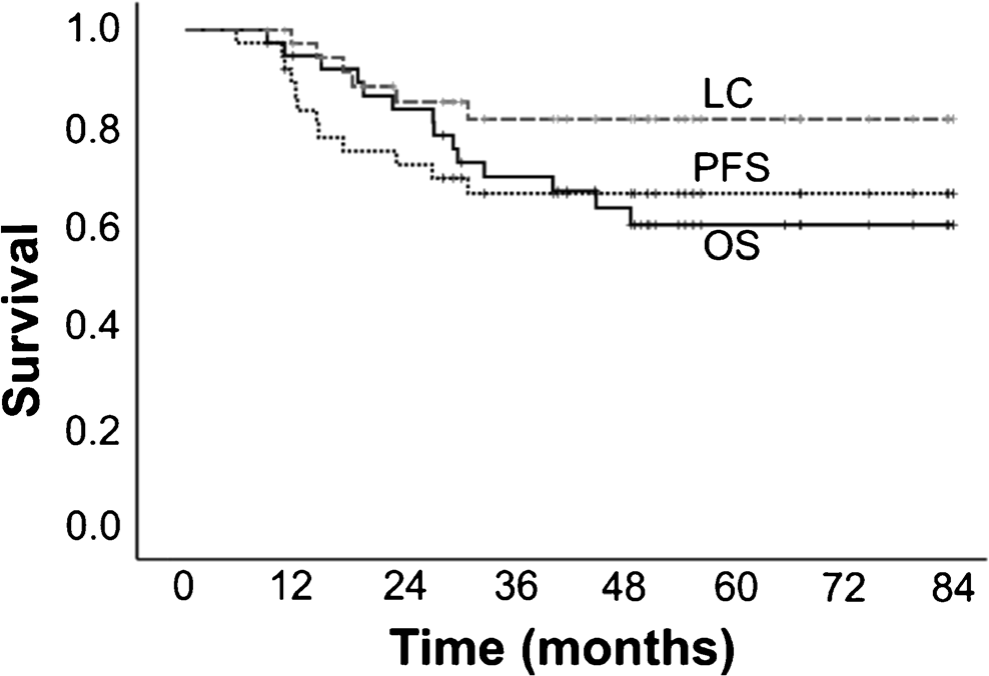
Overall survival (OS), progression free survival (PFS), and local control (LC) rates for NSCLC stage I patients treated with carbon-ion therapy (C-ion RT)

The 1-year/2-year/3-year OS, PFS, and LC rates in percentage were 95/84/71, 87/73/67, and 97/86/82, respectively (Fig. 1). Twelve patients showed recurrences: six had local recurrence, two manifested lymph node metastasis, one patient each showed multiple lung metastases or liver metastasis or brain metastasis (total three patients), and one patient exhibited brain and lymph node metastasis. Out of 14 deaths, six succumbed to lung cancer and eight died because of comorbidities. Two tumor-bearing survivors underwent repeat C-ion RT and 21 were disease-free survivors.

### PET parameters and characteristics of primary tumor

The median tumor size of the primary tumor was 22 mm (8.2–49). Median SUVmax, SUVpeak, and SUVmean were 4.57 (0.91–23.73), 3.59 (0.67–19.87), and 2.6 (0.59–14.49), respectively. Medians of volumetric parameters MTV and TLG were 2.16 (0–70.64) and 8.05 (0–393.9), respectively.

### Cox proportional hazard regression analyses

In the univariate analysis (Table 2), among the clinicopathological variables, only age and histology were significant prognostic factors for OS, whereas none of the variables proved to be significant for PFS. Among FDG PET/CT parameters analyzed as categorical variables, the significant prognostic factors for OS were MTV and TLG (HR = 5.2, *p* < 0.02) and PFS was MTV (4.6, *p* < 0.03). None of the variables were significant for LC.

**Table 2:** Univariate and Multivariate Analysis for OS and PFS with Cox Proportional Hazards Model

Univariate Analysis	OS			PFS		
Independent variable	HR	95% CI	*p*	HR	95% CI	*p*
Age	1.087	1.008, 1.172	0.030	1.009	0.942, 1.081	0.797
Male Sex	2.425	0.542, 10.846	0.247	4.762	0.614, 36.921	0.135
Tumor size (continuous, cm)	1.039	0.994, 1.087	0.093	1.033	0.984, 1.083	0.191
Tumor size (categorical; based on medium)	1.965	0.680, 5.675	0.212	2.230	0.706, 7.045	0.172
Stage IA	0.539	0.184, 1.580	0.260	0.547	0.164, 1.824	0.326
Adenocarcinoma	0.279	0.093, 0.834	0.022	0.697	0.220, 2.202	0.538
Irradiation dose (52.8 Gy)	1.094	0.944, 1.268	0.234	1.085	0.924, 1.273	0.320
Operability	2.764	0.967, 7.899	0.058	1.357	0.408, 4.515	0.619
Categorical FDG variables (based on median)						
SUVmax	2.373	0.794, 7.091	0.122	2.032	0.643, 6.425	0.227
SUVpeak	2.373	0.794, 7.091	0.122	2.032	0.643, 6.425	0.227
SUVaverage	2.373	0.968, 1.255	0.122	2.032	0.643, 6.425	0.227
MTV	5.199	1.447, 18.675	0.012	4.634	1.250, 17.179	0.022
TLG	5.199	1.447, 18.675	0.012	3.032	0.910, 10.105	0.071
Multivariate Analysis						
MTV	4.832	1.211, 19.274	0.026	5.302	1.316, 21.359	0.019
TLG	3.728	0.842, 16.510	0.083			
Age	1.092	1.001,1.190	0.048			

Multivariate analysis was performed by considering the significant univariate analysis results. In the multivariate analysis, age and MTV were found to be significant independent prognostic factors for OS with HR of 1.09 for age (*p* < 0.05) and HR of 4.8 for MTV (*p* < 0.03), respectively. For PFS, MTV was the only significant factor in multivariate analysis with HR of 5.3 (*p* < 0.02).

### Kaplan-Meier curves and log-rank test

**Fig. 2:**
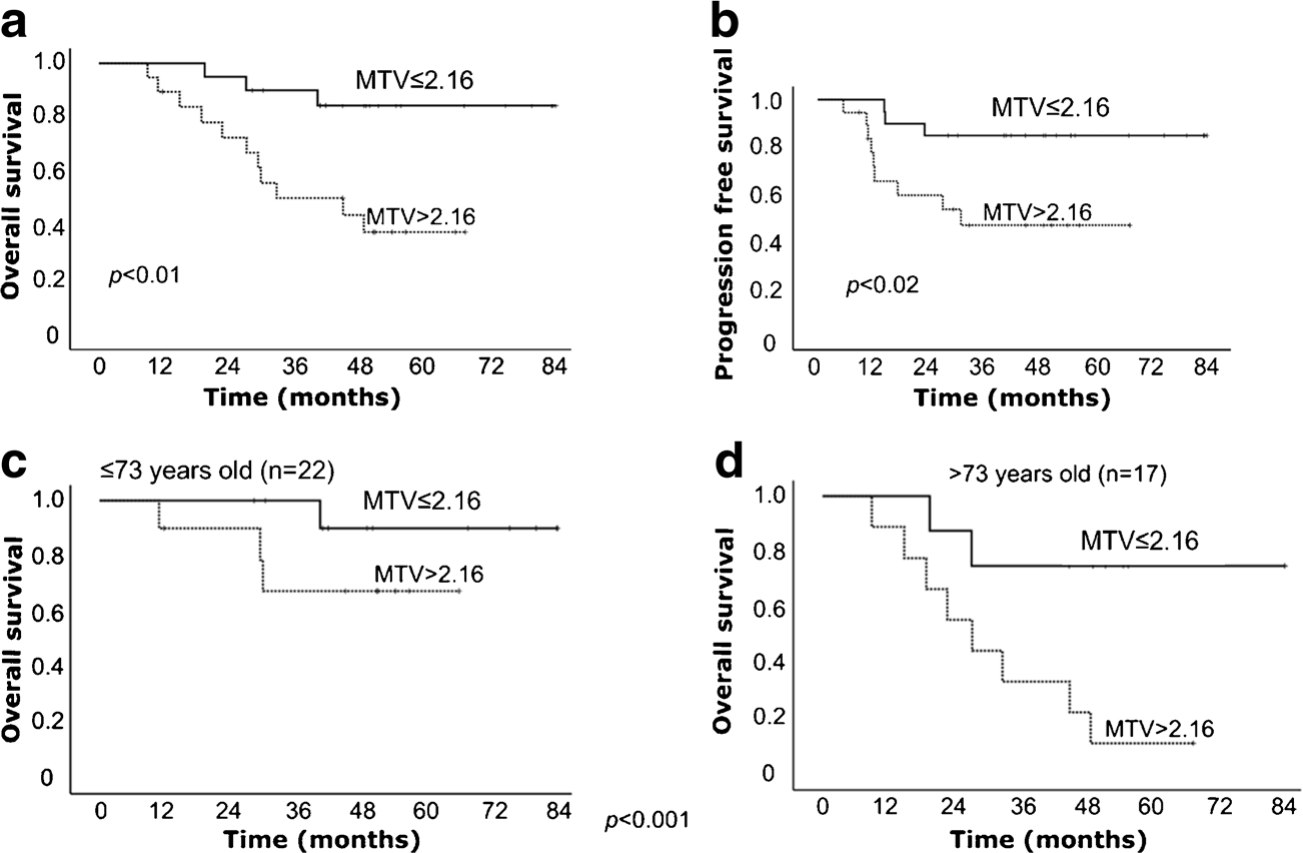
Kaplan-Meier curves of **a** OS and **b** PFS stratified by MTV, and **c**, **d** OS stratified by age-adjusted MTV in stage I NSCLC patients treated with C-ion RT

Kaplan-Meier curves showed significant differences in low and high MTV groups stratified by median MTV value for OS (*p* < 0.01) and PFS (*p* < 0.02; Fig. 2).

As age was also significant for OS, the Kaplan-Meier curve for MTV for OS was adjusted for the age, which was categorized based on median. Log-rank test of Kaplan-Meier curve for MTV adjusted for age showed significant results (*p* < 0.01).

### Assessment of predictive performance

Time-dependent ROC curve analysis showed MTV had the greatest accuracy among all FDG parameters for both OS (AUC = 0.74, *p* < 0.02) and PFS (0.72, *p* < 0.04). The AUC for tumor size was the lowest and insignificant compared to other parameters for OS (0.63, *p* > 0.1) and PFS (0.62, *p* > 0.2).

### Differences in the FDG PET/CT parameters based on histology 

**Fig. 3:**
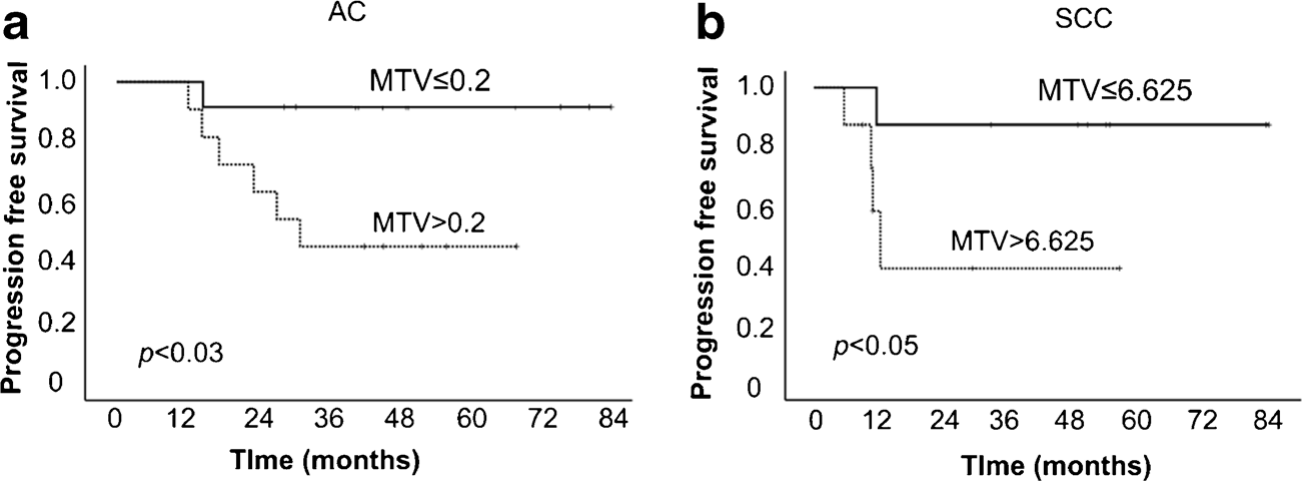
Kaplan-Meier curves of PFS stratified by MTV in **a** stage I lung adenocarcinoma S and **b** SCC treated with C-ion RT

The median MTVs of AC and SCC were 0.2 cm^3^ (range, 0–43.7 cm^3^) and 6.625 cm^3^ (0–70.6 cm^3^, *p* < 0.01), respectively. The Kaplan-Meier curves for PFS in AC and SCC using MTV of 2.16 cm^3^ as cut-off was found significant only for AC (*p* < 0.04) but insignificant in SCC (*p* > 0.2). However, the Kaplan-Meier curves for PFS using median values of MTV in both AC and SCC, 0.2 and 6.625 cm^3^ respectively, were found significant (*p* < 0.03 in AC and *p* < 0.05 in SCC, Fig. 3).

### Reliability and reproducibility of MTV compared to tumor size

Pearson correlation (r) and regression analysis between two reading times (intraobserver) and between two observers (interobserver) showed very high reproducibility for MTV (*r* = 0.998–1, intercept = 0.04–0.2%, slope = 0.98–1.04) and lowest for tumor size (*r* = 0.85–0.92, intercept = 1.39–3.99, slope = 0.76–0.99). Intraclass correlation coefficient (ICC) values were also high for MTV (ICC = 0.99–1) parameters compared to tumor size (ICC = 0.94–0.95).

## Discussion

So far we know this was the first clinical study to evaluate the prognostic significance and predictive ability of the volumetric FDG PET/CT parameters in stage I NSCLC treated by C-ion RT, and we found MTV was the only significant prognostic factor for OS and PFS.

We also evaluated the histological differences of MTV in risk stratification for survival outcome. MTV-based risk stratification has already been reported in locally advanced NSCLC, by Salavati et al. [15], but histology was not considered. Although a previous report of differences in glucose metabolism in AC and SCC exists [12], little attention has been paid for incorporating the differences between histology in prognostic studies [8–11].

In our subgroup analysis based on histology, the median values of MTV were significantly different between AC and SCC. This finding was in agreement with a previous report [12] that demonstrated significant differences in levels of GLUT1 expression in AC and SCC and proved the glucose metabolism differs in AC and SCC. In addition, the cut-off values of MTV based on median in AC and SCC in our study were found significant for PFS, while single cut-off based on overall cohort failed for SCC. They also reported that AC has high metastatic potential than SCC [12]. It means that even if MTV is low in AC, it bears higher metastatic potential compared with that in SCC. Therefore, different cut-offs may be required for AC and SCC to stratify the risks for survival outcome.

**Fig. 4:**
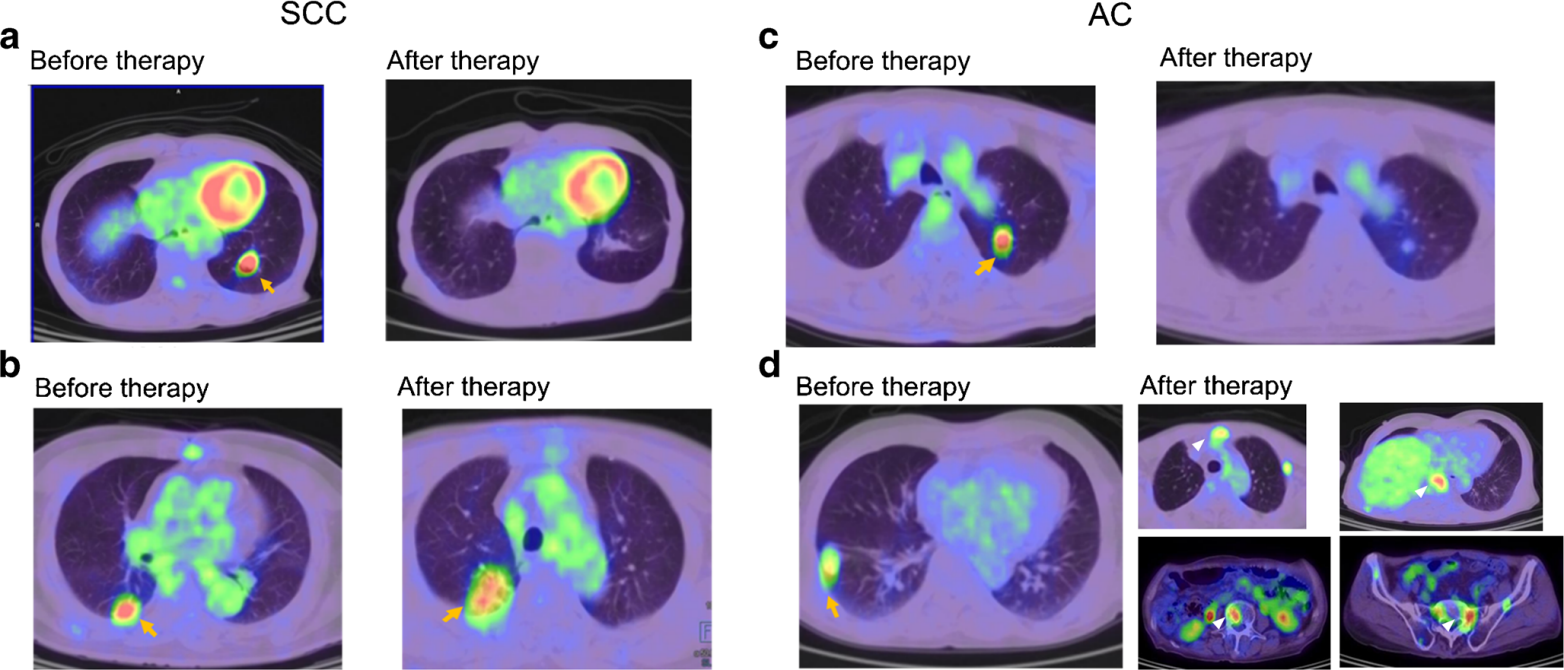
Axial FDG PET/CT fusion images of representative cases of stage I SCC and stage I AC with lung tumor lesions indicated by yellow arrows, and bone metastases indicated by white arrowheads. SCC patients: **a** 80-year-old male having tumor with SUVmax 10.34 and MTV 5.78 cm^3^, with 48.6 months PFS/OS, and **b** 76-year-old male having tumor with SUVmax 7.04 and MTV 7.96 cm^3^ with 5.5 months PFS and 27.1 months OS; AC patients: **c** 77-year-old male having tumor with SUVmax 5.85 and MTV 1.59 cm^3^ with 55.4 months PFS/OS, and **d** 73-year-old male having tumor with SUVmax 4.28 and MTV 2.51 cm^3^ with 17.2 months PFS and 29.7 months OS. Multiple bone metastases are observed in case “**d**,” shown by white arrowheads in after-therapy images; upper row—left: sternal, right: thoracic vertebra; lower row—left: lumbar vertebra, right: sacrum. In both SCC and AC, MTV correlated better than SUVmax with survival outcomes

Our study findings differed from previous study result that showed SUVmax was significant for survival outcome in C-ion RT–treated NSCLC stage I patients [11]. The representative cases show the differences in SUVmax and MTV in AC and SCC, and they demonstrate that MTV, not SUVmax, correlated well with the survival prognosis of the patients (Fig. 4). Low MTV despite high SUVmax value had better prognosis and vice versa. These findings were consistent with a report [10] demonstrating that volumetric parameters were better prognostic factors than SUVs, although we did not find TLG to be significant. By contrast, Satoh et al. observed SUVmax in addition to MTV and TLG were significant for PFS prognosis in < 3 cm NSCLC tumors treated with SBRT [9]. The reason for variable results in these studies could be due to the different methods used to obtain MTV. We used the best method proved among all previously, i.e., using SUV of 2.5 or more, which is least sensitive to uptake time and highly reproducibile, as reported by Kitao et al. [16]. Nonetheless, as mentioned earlier, none of these studies considered histologic differences in the PET parameters during risk stratification for the survival outcomes.

MTV was also the parameter to have highest AUC or accuracy in ROC curve and tumor size had the lowest. These findings are similar with the findings from a study conducted by Miyabe J. et al. demonstrating MTV to be superior than T-classification in larynx preservation approach in total laryngectomy after chemoradiotherapy [17].

Since the PET parameters are calculated in a software and are semi-quantitative [18], even young nuclear physicians or trainees can make reproducible results. Our findings showed MTV had high intra- and inter-observer agreement. In addition, Liu et al. have reported superior consistency of MTV over other FDG PET/CT parameters of NSCLC measured at two different tracer uptake times [19].

There are some limitations of the study. Firstly, the study design was retrospective and we had a small sample size. However, owing to the high cost of the treatment and limited treatment centers around the world, this study was crucial in understanding the survival analysis in C-ion RT–treated Stage I NSCLC in detail. This study provided some insight into the prognostic significance of the volumetric parameters in C-ion RT–treated NSCLC stage I patients. Next, it was a single-center study. Further multi-center prospective studies with larger patient cohorts are warranted to validate the results.

## Conclusion

Only MTV among the FDG PET/CT parameters was found to have independent prognostic significance with high reproducibility and reliability and superior to tumor size in the OS and PFS of NSCLC stage I treated with C-ion RT. Histology may also need to be considered in determining the cut-off for stratifying the low and high MTV groups.
